# Micron-Sized Thiol-Functional Polysilsesquioxane Microspheres with Open and Interconnected Macropores: Preparation, Characterization and Formation Mechanism

**DOI:** 10.3390/molecules29061204

**Published:** 2024-03-08

**Authors:** Lu Han, Zhenyu Nie, Rongsheng Gao, Zhengyang Jiang, Chengyou Kan

**Affiliations:** Key Laboratory of Advanced Materials of Ministry of Education, Department of Chemical Engineering, Tsinghua University, Beijing 100084, China

**Keywords:** polysilsesquioxane, micron-sized microsphere, open macropore, thiol, phase separation

## Abstract

Polysilsesquioxane (PSQ) microspheres have shown promise in many fields, but previous studies about porous PSQ microspheres are scarce. Herein, we fabricated novel micron-sized thiol-functional polysilsesquioxane (TMPSQ) microspheres with open and interconnected macropores by combining inverse suspension polymerization with two-step sol–gel and polymerization-induced phase separation processes, without using phase-separation-promoting additives or sacrificial templates. The chemical composition of the TMPSQ microspheres was confirmed using FTIR and Raman spectroscopy. The morphology of the TMPSQ microspheres was characterized using SEM and TEM. TGA was employed to test the thermal stability of the TMPSQ microspheres. Mercury intrusion porosimetry and nitrogen adsorption–desorption tests were performed to investigate the pore structure of the TMPSQ microspheres. The results showed that the TMPSQ microspheres had open and interconnected macropores with a pore size of 839 nm, and the total porosity and intraparticle porosity reached 70.54% and 43.21%, respectively. The mechanism of porous generation was proposed based on the morphological evolution observed using optical microscopy. The macropores were formed through the following four steps: phase separation (spinodal decomposition), coarsening, gelation, and evaporation of the solvent. The macropores can facilitate the rapid mass transfer between the outer and inner spaces of the TMPSQ microspheres. The TMPSQ microspheres are promising in various fields, such as catalyst supports and adsorbents.

## 1. Introduction

Polysilsesquioxane (PSQ) microspheres are a type of spherical organic–inorganic material that has captured the attention of researchers in many areas. With inorganic –Si–O–Si– networks and organic side groups, PSQ microspheres exhibit unique and valuable features, such as excellent mechanical properties, chemical stability, biocompatibility, and low dielectric constants [1]. Therefore, PSQ microspheres have been recognized as a promising material in various applications. They are excellent pollutant adsorbents [2], bioimaging agents [3], chromatographic stationary phases [4], modifiers [5], and light scattering agents [6].

Porous materials have always been research hotspots in recent years because of their high surface area, low density, tunable pore size and volume, and more active sites on the surface. They have been applied in the fields of drug delivery [7], catalyst support [8], energy storage [9], adsorption [10], separation [11,12], etc. Among different types of pores, macropores have the advantage of easy and rapid mass transfer along pore channels, which can promote the interaction between the exterior substances and the active sites in materials [13,14]. However, introducing pore structures, especially macropores, into some functional materials is still a challenge.

Compared with nonporous PSQ microspheres, studies on porous PSQ microspheres are still relatively few. Different from silica microspheres, PSQ microspheres have relatively vulnerable organic side groups. Therefore, methods with fierce steps or conditions are unsuitable for the preparation of porous PSQ microspheres. For example, Bai et al. [15] utilized a porous organic polymer crosslinked microsphere as a sacrificial hard template to synthesize porous silica microspheres, and the template was removed using calcination at 600 °C for 10 h. At such a high temperature, organic side groups are not retained in PSQ microspheres [16]. In addition, the calcination process causes an increase in costs and production complexity, and the pore structure could also be damaged [17]. Thus, to prepare porous PSQ microspheres, mild methods should be applied under mild conditions. Recently, some PSQ microspheres with inherent mesopore structures have been reported [18]. They were synthesized without using structure-directing agents, but the surface area and pore volume are relatively low. Even though an alkali-heat treatment can enlarge the pore volume and surface area of such mesopore structures, the pore size is still less than 10 nm [4]. Cationic surfactants can also introduce mesopores into PSQ microspheres, yet the pore size is also relatively small [19]. Moreover, the W/O/W emulsion system has also been used to fabricate porous PSQ microspheres [17]. The micron or submicron cavities were caused by internal-phase droplets, but those cavities were generally separated and enclosed. In this situation, mass transfer among different cavities of microspheres and external space is limited. Johnston et al. [20,21] prepared macroporous poly(3-mercaptopropylsilsesquioxane) microspheres via an O/W-emulsion-based two-step sol–gel method, and most of the macropores were closed. Han et al. [22] prepared macroporous polymethylsilsesquioxane microspheres via microfluidic technology, and the shells of the microspheres were thin and dense films, which could also prevent mass transfer. To the best of our knowledge, there is hardly any research on functional PSQ microspheres with open and interconnected macropores prepared by a facile and mild method.

The phase separation phenomena that occurred in the sol–gel process of alkoxysilanes have been investigated in recent decades [23,24,25]. Through polymerization-induced phase separation, silica and polysilsesquioxane monoliths with interconnected macropores can be prepared [26,27,28]. Such a phase separation method was also employed to obtain silica-based macroporous microspheres [11,12,13,29,30,31]. The microspheres were fabricated using W/O systems, after dispersing the sol of tetraethyl orthosilicate and other alkoxysilanes into a non-polar solvent. However, in most cases, organic polymer additives were needed to induce phase separation, and were then eliminated by calcinating. Therefore, macroporous PSQ microspheres have not yet been successfully prepared through the polymerization-induced phase separation method. It should be noted that the above microspheres were prepared by one-step acid-catalyzed sol–gel processes under heating conditions. Compared with the one-step method, the two-step acid–base-catalyzed method can separate the hydrolysis and condensation reactions, which is beneficial for better controlling the complex sol–gel processes under mild conditions [32]. Yet the two-step method accompanied by polymerization-induced phase separation has not been used to produce functional PSQ microspheres with open and interconnected macropores.

In this work, novel micron-sized thiol-functional polysilsesquioxane (TMPSQ) microspheres with open and interconnected macropores were successfully prepared via a new synthetic strategy. This strategy combined inverse suspension polymerization with two-step sol–gel and polymerization-induced phase separation processes. The disperse phase consisted of methyltrimethoxysilane (MTMS) and (3-mercaptopropyl)trimethoxysilane (MPTMS) as precursors, the mixture of water and methanol (MeOH) as solvents, and HCl (aq) and NH_4_OH (aq) as catalysts. The continuous phase was made up of Span 80 and liquid paraffin. No further additive or template was utilized, and no other post-treatment process except washing and drying was employed. The morphology, chemical composition, thermal stability, and pore structure of te TMPSQ microspheres were investigated. The morphological evolution of the TMPSQ microspheres was monitored, and the formation mechanism of the open and interconnected macropores was demonstrated.

## 2. Results and Discussion

### 2.1. Preparation of TMPSQ Microspheres

Phase separation refers to a thermodynamic process in which a homogeneous phase is converted to two separated phases spontaneously, based on the minimized Gibbs free energy principle. Spinodal decomposition is a type of phase separation that happens in the unstable region of the miscibility window in an equilibrium phase diagram. After spinodal decomposition, the system turns into two distinct conjugated phases with a bicontinuous morphology [33]. In the sol–gel processes of alkoxysilanes, the two conjugated phases are the gel-rich phase and the solvent-rich phase [23]. Herein, the solvent refers to the mixture of water and polar solvents such as methanol, ethanol, and formamide. The interconnected structure caused by spinodal decomposition is eventually frozen by the gelation of systems. After eliminating the solvent, macroporous materials are obtained.

The Flory–Huggins equation (Equation (1)) can be applied to demonstrate the phase separation tendency as follows [34,35]:

(1) $$\Delta G=\Delta H-T\Delta S\propto \mathrm{RT}(\chi_{\mathrm{AB}}\Phi_{A}\Phi_{B}+\frac{\Phi_{A}}{x_{A}}\ln\Phi_{A}+\frac{\Phi_{B}}{x_{B}}\ln\Phi_{B})$$

where Δ*G*, Δ*H*, and Δ*S* are the Gibbs free energy change, enthalpy change, and entropy change of mixing, respectively; *R* refers to the gas constant; *T* is the temperature; *χ*_AB_ denotes the interaction parameter between the components (the higher the value, the larger the difference and the lower the compatibility between the two components); and *Φ*_A_, *Φ*_B_, *x*_A_, and *x*_B_ are the volume fractions and polymerization degrees of two varied components (oligomer/polymer and solvent, respectively). In silicon-based sol–gel systems, with the ongoing condensation reactions, the hydrophilic silanol groups are consumed and become less hydrophilic Si–O–Si. The polymerization degree of oligomers increases and the compatibility between oligomers and solvent decreases, which results in the increase of *x*_A_ and *χ*_AB_, and thus Δ*H* increases and Δ*S* decreases. Therefore, the increase in Δ*G* becomes the driving force for phase separation.

In tetraalkoxysilane–silica systems, organic water-soluble polymers, such as poly(sodium styrenesulfonate) [23], poly(acrylic acid) [36], and poly(ethylene oxide) [37], are generally added to the systems in most cases, which can induce phase separation. In organotrialkoxysilane–polysilsesquioxane systems, due to the hydrophobic organic side groups, the oligomers become less compatible with the solvent, and the phase separation tendency becomes stronger [38]. For example, in a MTMS–MeOH–H_2_O–HNO_3_ system, polymethylsilsesquioxane monoliths with interconnected macropores were synthesized without any additives, which made the preparation and post-treatment procedures simpler [39]. However, the formation of macroporous structures highly depends on the hydrophobicity of the side groups of precursors. With a longer carbon chain of side groups, the hydrophobicity of MPTMS is higher than that of MTMS, which can cause a much higher phase separation tendency [40]. In this case, macroscopic double phases rather than a homogeneous gel with two microscopic interconnected phases were formed [39]. Therefore, MTMS was selected as a co-precursor to limit the excessive phase separation tendency caused by MPTMS.

**Figure 1:**
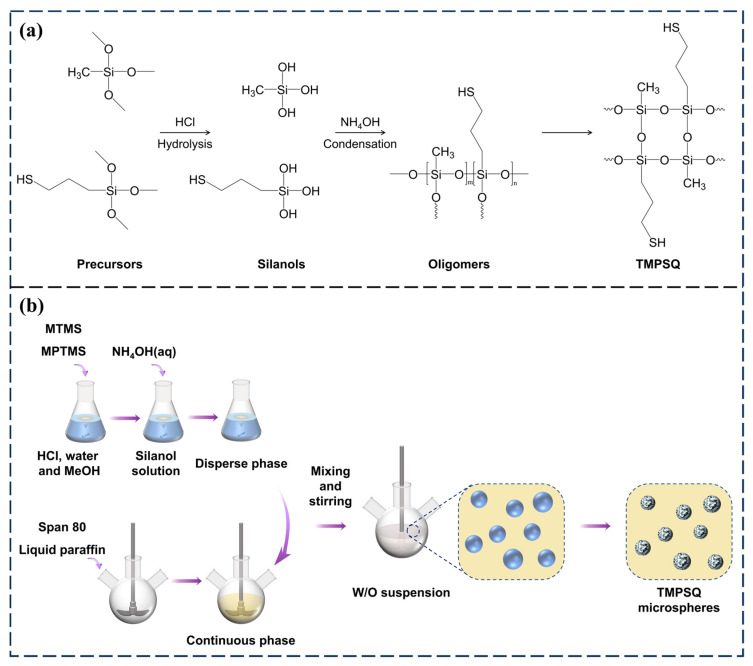
(**a**) Synthetic reactions for TMPSQ, and (**b**) a schematic illustration of the synthetic procedure for TMPSQ microspheres.

In this study, the TMPSQ was synthesized via an acid–base-catalyzed two-step method, and the illustration of chemical reactions is shown in Figure 1a. In the disperse phase, the reactions of precursors were catalyzed by HCl and NH_4_OH successively. After being mixed with HCl (aq) and MeOH, the precursors were hydrolyzed to soluble silanols. Since the condensation reaction is slow under an acidic condition [41], the sol–gel transition did not happen. After the addition of NH_4_OH (aq), the condensation reaction was accelerated, and the silanols became oligomers. With the increase in polymerization degree and the decrease in compatibility with the solvent, the oligomers were separated from the sol. After a period of time, the crosslinked network was established throughout the disperse phase, and the TMPSQ gels were formed. The results showed that it took about 20 min for the disperse phase to turn into gels, and the rapid gelation could promptly freeze the interconnected morphology and avoid further phase separation [32].

After adding NH_4_OH (aq), the disperse phase was quickly dispersed into the continuous phase to form an inverse suspension polymerization system. As illustrated in Figure 1b, the disperse phase was separated into numerous spherical droplets, which were stabilized by Span 80. The sol–gel process and phase separation took place within the droplets. After gelation, the droplets eventually solidified into TMPSQ microspheres with interconnected macropores. The formation details will be discussed in Section 2.6. Since no sacrificial template or organic polymer additive was used in this system, no other post-treatment processes such as calcination and extraction were needed except washing and drying.

### 2.2. Morphology of the TMPSQ Microspheres

**Figure 2:**
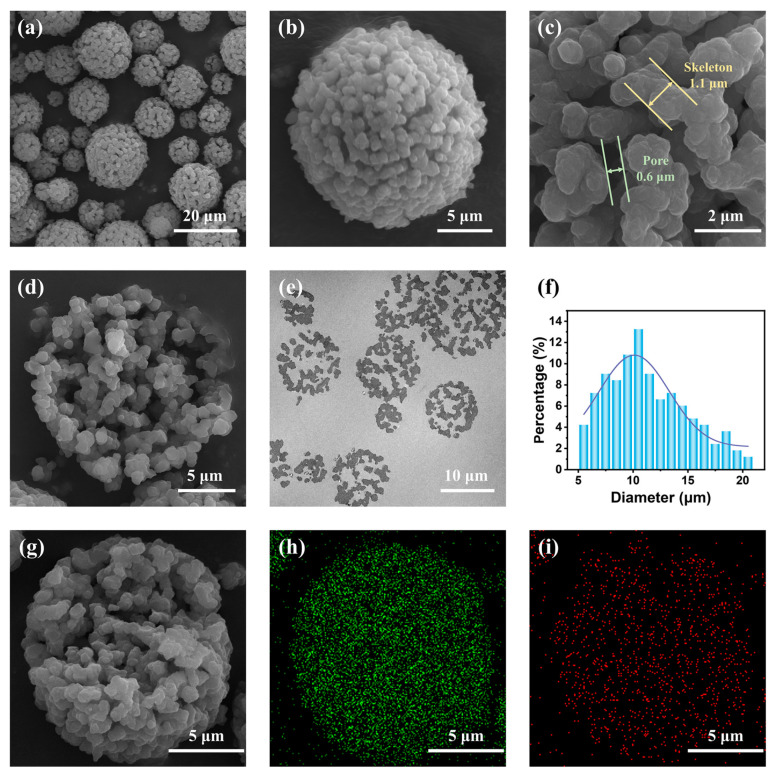
(**a**–**c**) SEM images of the TMPSQ microspheres at different magnifications, (**d**) SEM micrograph showing the inner structure of one TMPSQ microsphere, (**e**) TEM photograph of a microtomed slice of the TMPSQ microspheres, (**f**) size distribution histogram of the TMPSQ microspheres, and (**g**–**i**) EDS mapping images of one TMPSQ microsphere.

According to Figure 2a–e, the TMPSQ microspheres exhibited a spherical structure with observable submicron-sized skeletons and large pores. The widths of skeletons and macropores were measured to be 1.0–1.2 μm and 0.6–0.9 μm, respectively. The skeletons and pores were continuous and interwoven with each other, which was similar to the typical bicontinuous structure formed by spinodal decomposition. Moreover, the macropores had an opening structure, meaning that the internal space of microspheres was connected to the outer space. The unique pore structure could provide enough wide tunnels for fast mass transfer. As can be seen from the EDS elemental mapping images in Figure 2g–i, Si and S elements were uniformly distributed on the skeletons of the TMPSQ microsphere, both on the surface and in the inner part. The size distribution diagram in Figure 2f suggested that the TMPSQ microspheres were of unimodal distribution. The numerical average diameter (*D*_average_) and the coefficient of variation (CV) of the TMPSQ microspheres were 11.35 μm and 32.5%, respectively.

### 2.3. Composition of the TMPSQ Microspheres

**Figure 3:**
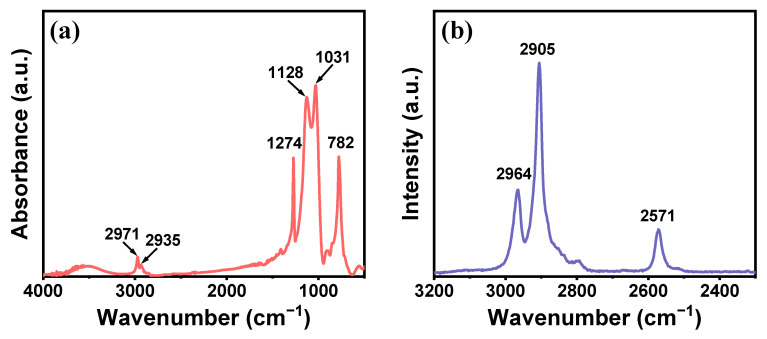
(**a**) FTIR and (**b**) Raman spectra of the TMPSQ microspheres.

FTIR and Raman analyses were employed to characterize the chemical composition of the TMPSQ microspheres. As exhibited in Figure 3a, the strong bands at 1031 cm^−1^ and 1128 cm^−1^ in the FTIR spectrum were attributed to asymmetric stretching vibrations of Si–O–Si in the skeletons of the TMPSQ microspheres [17]. The organic parts can be confirmed by the stretching vibration bands of Si–C at 782 cm^−1^ [42], the symmetrical deformation vibration band of CH_3_ at 1274 cm^−1^, and the stretching vibration bands of C–H in CH_3_ and CH_2_ at 2971 cm^−1^ and 2935 cm^−1^, respectively [2]. Since the dosage of MPTMS was less than that of MTMS, the weak characteristic band attributed to thiol groups was insignificant in the FTIR spectrum, but the proof of the presence of thiol groups can still be found in the Raman spectrum (Figure 2b), which was the stretching vibration band of S–H at 2571 cm^−1^ [4]. Moreover, bands at 2964 cm^−1^ and 2905 cm^−1^ in the Raman spectrum belonged to the antisymmetric stretching vibration and symmetric stretching vibration of C–H in CH_3_, respectively, which further suggested the existence of methyl groups [43].

### 2.4. Thermal Stability of the TMPSQ Microspheres

**Figure 4:**
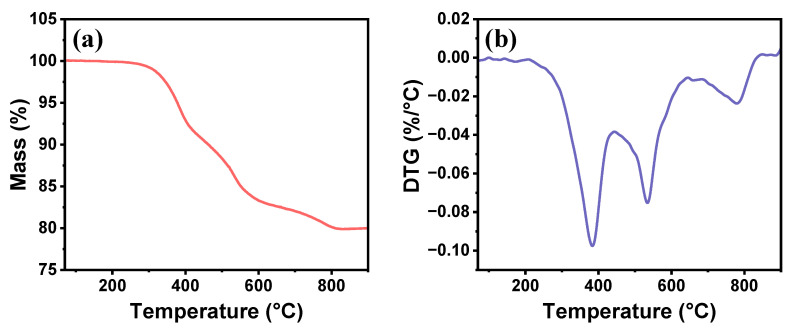
(**a**) TGA and (**b**) derivative thermogravimetry (DTG) curves of the TMPSQ microspheres.

The thermal stability of the TMPSQ microspheres was tested using TGA. As indicated in Figure 4, the microspheres were thermally stable under 210 °C. Three steps of weight loss can be observed in the TGA curve from 210 °C to 830 °C, and the total weight loss was 20.1%. The first step in the range of 210 °C to 440 °C showed a sharp weight loss (9.0%), which resulted from the decomposition of mercaptopropyl groups [44]. The weight losses in the second step ranging from 440 °C to 640 °C (8.1%) and the third stage ranging from 640 °C to 830 °C (2.8%) were attributed to the thermal decomposition of methyl groups [45], but they referred to different pyrolysis reactions. In the former temperature region (440–640 °C), SiO_2_ and Si(CH_3_)_4_ were obtained, whereas in the latter temperature region (640–830 °C), the products of pyrolysis were SiO_1_._5_C_0_._25_ and CH_4_ [46]. The TMPSQ microspheres had a similar decomposition temperature and thermal decomposition behavior to other PSQ microspheres, which remained stable after heat processing [16,47]. It can be inferred that the thermal stability of the TMPSQ microspheres can meet the requirements of potential applications such as adsorbents and catalyst carriers.

### 2.5. Pore Structure of the TMPSQ Microspheres

**Figure 5:**
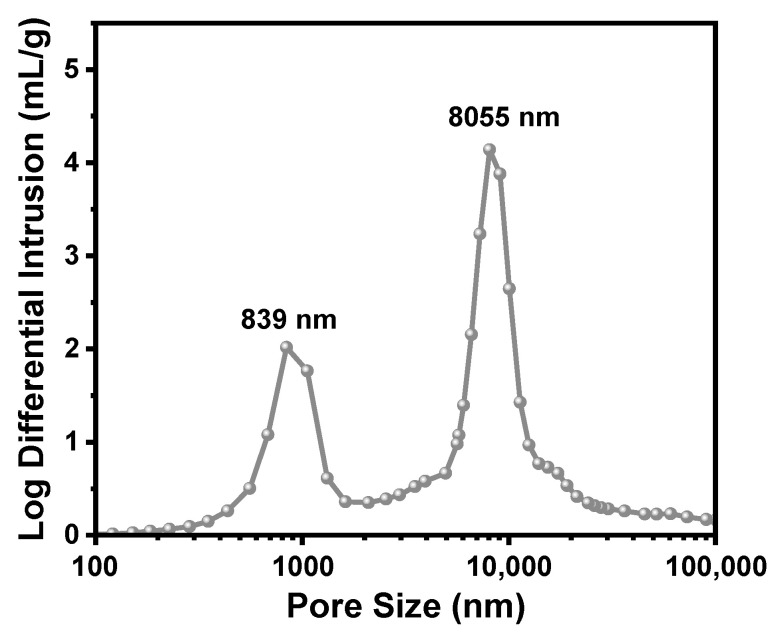
Pore size distribution curve of the TMPSQ microspheres determined using mercury intrusion porosimetry.

Mercury intrusion porosimetry was conducted to characterize the macropore structures of TMPSQ microspheres. For micron-sized porous microspheres, the pore size distribution is generally bimodal, due to the coexistence of intraparticle pores and interparticle spaces [48]. As presented in Figure 5, the TMPSQ microspheres displayed a bimodal pore size distribution, with two peaks at 839 nm and 8055 nm, respectively. Based on the aforementioned discussion on the morphology of TMPSQ microspheres in Section 2.2, it can be speculated that the peak at 839 nm corresponded to the macropores within the microspheres, and the peak at 8055 nm was attributed to the interparticle spaces. The mercury intrusion data showed that the total pore volume (*V*_pore_) was 2.23 mL/g, and the total porosity (*P*_total_) was 70.54%.

In the bimodal pore size distribution curve (Figure 5), the minimal point between the two peaks in the pore size distribution curve was considered to be the division point between interparticle voids and intraparticle pores. The volume of interparticle voids (*V*_inter_) and the volume of intraparticle pores (*V*_intra_) were 1.52 mL/g and 0.71 mL/g, respectively, and the intraparticle porosity (*P*_intra_) was calculated to be 43.21%. These results confirmed the considerable macroporosity of the TMPSQ microspheres.

**Figure 6:**
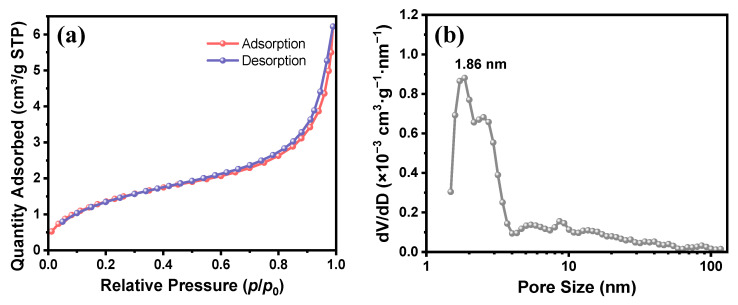
(**a**) Nitrogen adsorption and desorption isotherms and (**b**) NLDFT pore size distribution curve of the TMPSQ microspheres.

Nitrogen adsorption–desorption analysis was performed on the TMPSQ microspheres to further characterize the micropore and mesopore structures. The pore volume and BET surface area were 0.0096 cm^3^/g and 5.23 m^2^/g, respectively. As shown in Figure 6a, the nitrogen adsorption–desorption isotherms showed type II characters, indicating that the TMPSQ microspheres were macroporous materials [49]. Moreover, a narrow type H3 hysteresis loop with a very small area was observed in the isotherms, suggesting that the TMPSQ microspheres had a poor mesopore structure in the skeletons. According to the NLDFT pore size distribution curve presented in Figure 6b, no micropore existed, and the mesopores were centered at 1.86 nm. The mesopores are formed probably due to the microphase separation, and a small amount of solvent remains inside the skeletons of TMPSQ microspheres [50]. After drying, both the large quantity of solvent between skeletons and the small amount of solvent inside the skeletons were eliminated, and hence the TMPSQ microspheres with obvious macropores and insignificant mesopores were formed.

### 2.6. Morphological Evolution of the TMPSQ Microspheres

**Figure 7:**
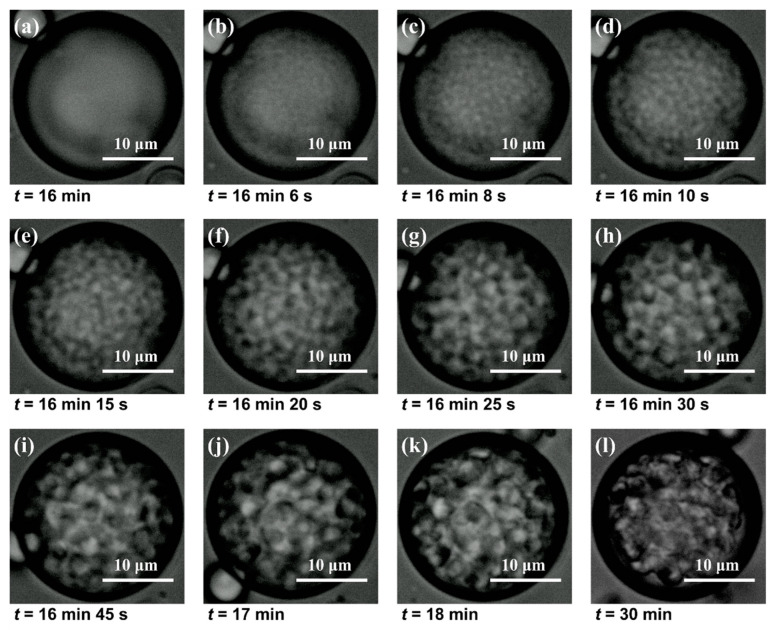
Optical microscopic images of one disperse phase droplet in the reaction system after adding NH_4_OH (aq) for different times: (**a**) 16 min, (**b**) 16 min 6 s, (**c**) 16 min 8 s, (**d**) 16 min 10 s, (**e**) 16 min 15 s, (**f**) 16 min 20 s, (**g**) 16 min 25 s, (**h**) 16 min 30 s, (**i**) 16 min 45 s, (**j**) 17 min, (**k**) 18 min, and (**l**) 30 min.

The morphological evolution of the TMPSQ microspheres was investigated using an optical microscope, and the time-resolved microscopic photos are displayed in Figure 7. Considering that the phase separation and sol–gel transition primarily depend on the addition of an alkali catalyst, the time to add NH_4_OH (aq) to the disperse phase was set to be the starting time (*t* = 0). Initially, there was a homogeneous solution inside the disperse phase droplet, and no morphological change of the droplet was observed before *t* = 16 min (Figure 7a). At this stage, silanols were gradually converted into oligomers via the condensation reaction, but the degree of polymerization was relatively low, and thus the oligomers were still dissolved in the disperse phase. With the ongoing condensation reaction, the degree of polymerization further increased, and the compatibility between oligomers and solvent decreased. As a result, the Δ*G* of the system inside the disperse phase increased to positive, which facilitated the phase separation. As can be seen in Figure 7b, at 16 min 6 s, a network structure with bright and dark parts suddenly appeared, suggesting the occurrence of spinodal decomposition. Then, both bright and dark domains were quickly widened within 24 s (Figure 7c–h), indicating that the coarsening process was ongoing. Here, coarsening denoted the growth of domains during phase separation, which was driven by interfacial energy and achieved by the diffusion or migration of compositions [51]. After that, the domains were still slowly enlarged in the following 90 s, and the skeletons of the microsphere gradually became clear (Figure 7i–k). In this period, since the condensation reaction continued, the crosslinking degree and viscosity of oligomers increased, which limited the diffusion and migration of oligomers in the droplet, and the droplet turned into a spherical gel particle. The condensation of the residual silanols or low-crosslinked oligomers might be responsible for the slow expansion of skeletons. Finally, the interconnected structure inside the droplet was fixed after gelation (Figure 7l), and the TMPSQ microsphere was formed.

**Figure 8:**
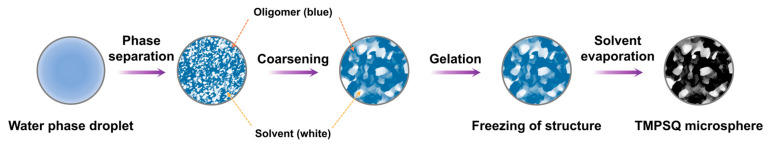
Schematic illustration of the structural evolution of a TMPSQ microsphere.

The formation mechanism of macropores in a TMPSQ microsphere is illustrated in Figure 8, according to the above results and discussion. In the first stage, with the ongoing condensation reaction and the growing degree of polymerization of oligomers, the initially homogeneous silanol and oligomer solution in the disperse phase droplet is separated into two conjugated phases, which are the oligomer phase and solvent phase, respectively. In the second stage, both phases are enlarged through the coarsening process. In the third stage, the gelation process freezes the phase separation structure, and the coarsening process is terminated. After the removal of the solvent phase by washing and drying, macropores are left in the TMPSQ microsphere.

## 3. Materials and Methods

### 3.1. Materials

MTMS (AR) and MPTMS (AR) were supplied by 3A Chemicals Scientific Co., Ltd. (Shanghai, China). MeOH (AR), ethanol (AR), and cyclohexane (AR) were purchased from Shanghai Titan Scientific Co., Ltd. (Shanghai, China). Hydrochloric acid solution (35–37 wt%) and ammonia solution (25–28 wt%) were obtained from Modern Oriental Fine Chemistry Co., ltd. (Beijing, China). Liquid paraffin (AR, 0.83–0.86 g/mL of density, ≥300 °C of distillation temperature at atmospheric pressure) and Span 80 (CP) were supplied by Sinopharm Group Chemical Reagent Co., Ltd. (Shanghai, China). The chemicals were directly used without further purification. Water was deionized using a RO DI digital water purification system (Shanghai, China).

### 3.2. Preparation of TMPSQ Microspheres

Continuous phase: a total of 4 g of Span 80 and 76 g of liquid paraffin were added to a three-necked 250-mL flask equipped with a reflux condenser and an electric stirrer (HD2004W, Shanghai Sile Co., Ltd., Shanghai, China), and the mixture was stirred for 30 min at 300 rpm at 25 °C.

Disperse phase: a total of 3.41 g of MTMS, 0.98 g of MPTMS, 2.88 g of MeOH, and 2.30 mL of 0.01 M HCl (aq) were first mixed in a 50-mL round-bottom flask on a magnetic stirrer (MS-H280-Pro, SCILOGEX, Rocky Hill, CT, USA) for 2 h at 300 rpm at 25 °C. Then, 2.30 mL of 2 M NH_4_OH (aq) was dropwise added to the solution under stirring.

The disperse phase was quickly added to the continuous phase under 500 rpm of stirring, and the system remained under stirring for 16 h at 25 °C. After centrifugation at 5000 rpm for 5 min (GL-20G-II, Anting Instrument Factory, Shanghai, China), the acquired solid was washed with cyclohexane twice and with ethanol twice, and dried at 40 °C for 48 h to obtained TMPSQ microspheres.

### 3.3. Characterization of TMPSQ Microspheres

Field-emission scanning electron microscopy (SEM, ULTRA 55, ZEISS, Jena, Germany) was used to observe the surface morphology and internal structure of the TMPSQ microspheres with an accelerating voltage of 15 kV. The internal structure information of the TMPSQ microspheres was obtained by grinding the microspheres, and characterized using SEM. Equipped with the SEM, energy dispersive spectrometry (EDS, Oxford Instruments, Abingdon, UK) was applied to analyze the element distribution. All samples were coated with a thin platinum film before observation. The diameters of TMPSQ microspheres were measured using Nano Measurer 1.2 (Fudan University, Shanghai, China) based on the SEM images. By calculating the diameters of 200 particles, the numerical average diameter (*D*_average_) and standard deviation (*σ*) were calculated. To evaluate the polydispersity of the TMPSQ microspheres, the coefficient of variation (CV) was obtained according to Equation (2), where the smaller the value, the higher the uniformity:

(2) $$\mathrm{CV}=\frac{\sigma }{D_{\mathrm{average}}}\times 100\%$$

Transmission electron microscopy (TEM, Hitachi, H-7650B, Tokyo, Japan) was employed to obtain the cross-section micrograph of the TMPSQ microspheres. Before observation, the microspheres were first embedded in epoxy resin. After solidification, the composite was cut using an ultra-microtome machine (EM UC6, Leica, Wetzlar, Germany), and the thin slice (thickness 60–70 nm) was placed on a copper grid. The TEM analysis was performed on the above copper grid at an accelerating voltage of 80 kV.

Fourier-transform infrared (FTIR) spectroscopy was conducted on a Nicolet 560 spectrometer (Thermo Fisher Scientific, Waltham, MA, USA) using the KBr tablet method.

The Raman spectrum was recorded on an HR800 micro confocal Raman spectrometer (Horiba JobinYvon, Paris, France). The excitation wavelength was 633 nm.

Thermogravimetric analysis (TGA) was carried out on a TG 209 thermogravimetric analyzer (NETZSCH Scientific Instruments Trading (Shanghai) Ltd., China) in the range of 35–900 °C in a nitrogen atmosphere (20 mL/min flow rate), and the heating rate was 10 °C/min.

Mercury intrusion porosimetry (MIP) was employed to characterize the pore size distribution, total pore volume (*V*_pore_), and total porosity (*P*_total_) of the TMPSQ microspheres, using an AutoPore V 9620 analyzer (Micromeritics, Norcross, GA, USA) operated at a pressure range of 0.001 to 410 MPa. Both the advancing contact angle and the receding contact angle were 130°. The sample was dried in a vacuum drying oven (DZF-6020, Shanghai Honghua Instruments, Shanghai, China) at 100 °C for 2 h before analysis. The volume of interparticle voids (*V*_inter_) and the volume of intraparticle pores (*V*_intra_) were obtained from the cumulative intrusion volume data. The total volume of microspheres (*V*_microsphere_) was the volume of skeletons and intraparticle pores, which was calculated using Equation (3):

(3) $$V_{\mathrm{microsphere}}=V_{\mathrm{sample}}-V_{\mathrm{inter}}=\frac{V_{\mathrm{pore}}}{P_{\mathrm{total}}}-V_{\mathrm{inter}}$$

where *V*_sample_ stood for the total volume of the sample in the test. The intraparticle porosity (*P*_intra_) was then calculated according to Equation (4):

(4) $$P_{\mathrm{intra}}=\frac{V_{\mathrm{intra}}}{V_{\mathrm{microsphere}}}\times 100\%$$

Nitrogen adsorption and desorption isotherms of the TMPSQ microspheres were measured at 77.35 K using an ASAP 2460 adsorption analyzer (Micromeritics, USA). The Brunauer–Emmett–Teller (BET) method was applied to determine the specific surface area. The pore volume was obtained from the adsorption at the relative pressure (*p*/*p*_0_) of 0.99. The pore size distribution was determined via the method of nonlinear density functional theory (NLDFT). Before measurement, the sample was outgassed at 200 °C for 6 h under a high-vacuum condition.

The time-resolved morphology of a TMPSQ microsphere was carried out using a WMP-6508 microscope (Wumo Optical Instruments, Shanghai, China). After adding the disperse phase to the continuous phase, a drop of the suspension was placed on a slide, and then the sample was covered with a piece of cover slip. Finally, the sample was in situ observed under a brightfield condition.

## 4. Conclusions

Using MTMS and MPTMS as precursors, TMPSQ microspheres were successfully synthesized by combining the inverse suspension polymerization with two-step sol-gel and polymerization-induced phase separation processes. In this method, no template or additive was used, and no fierce post-treatment process was needed to form macropores in the TMPSQ microspheres. The microspheres were of unimodal distribution and high sphericity, with 11.35 μm of *D*_average_. The macropores inside the TMPSQ microspheres were open and interconnected, and the pore size was 839 nm. The TMPSQ microspheres showed a considerable porosity. *P*_total_ and *P*_intra_ were 70.54% and 43.21%, respectively. In the skeletons of TMPSQ microspheres, there still existed a poor mesopore structure (1.86 nm) resulting from the tiny remaining solvent. The generation of macropores was caused by spinodal decomposition that formed oligomer and solvent phases, coarsening that widened the two phases, gelation that fixed the interconnected structure, and the evaporation of the solvent. The TMPSQ microspheres have the advantages of easy separation, large specific surface area, and fast mass transfer, and have broad application prospects as catalyst supports and adsorbents.

## Data Availability

Data are contained within the article.
